# *In Vitro* Evolution of Bovine Foamy Virus Variants with Enhanced Cell-Free Virus Titers and Transmission

**DOI:** 10.3390/v7112907

**Published:** 2015-11-11

**Authors:** Qiuying Bao, Michaela Hipp, Annette Hugo, Janet Lei, Yang Liu, Timo Kehl, Torsten Hechler, Martin Löchelt

**Affiliations:** 1 Division of Molecuar Diagnostics of Oncogenic Infections, Research Focus Infection, Inflammation and Cancer, German Cancer Research Center (Deutsches Krebsforschungszentrum, DKFZ), Im Neuenheimer Feld 242, 69120, Germany; q.bao@dkfz.de (Q.B.); m.hipp@dkfz.de (M.H.); a.hugo@dkfz.de (A.H.); janet.lei@oncology.ox.ac.uk (J.L.); liuy18@nih.gov (Y.L.); t.kehl@dkfz.de (T.K.); t.hechler@hdpharma.com (T.H.); 2 Department of Oncology, University of Oxford, Oxford OX3 7DQ, UK; 3 Department Viral Recombination, HIV Dynamics and Replication Program, Center for Cancer Research, National Cancer Institute at Frederick, Frederick, MD 21702-1201, USA; 4 Heidelberg Pharma GmbH, 68526 Ladenburg, Germany

**Keywords:** bovine foamy virus, retrovirus, particle release, virus budding, Gag myristoylation, virus transmission

## Abstract

Virus transmission is essential for spreading viral infections and is a highly coordinated process which occurs by cell-free transmission or cell–cell contact. The transmission of Bovine Foamy Virus (BFV) is highly cell-associated, with undetectable cell-free transmission. However, BFV particle budding can be induced by overexpression of wild-type (wt) BFV Gag and Env or artificial retargeting of Gag to the plasma membrane via myristoylation membrane targeting signals, closely resembling observations in other foamy viruses. Thus, the particle release machinery of wt BFV appears to be an excellent model system to study viral adaption to cell-free transmission by *in vitro* selection and evolution. Using selection for BFV variants with high cell-free infectivity in bovine and non-bovine cells, infectivity dramatically increased from almost no infectious units to about 10^5^–10^6^ FFU (fluorescent focus forming units)/mL in both cell types. Importantly, the selected BFV variants with high titer (HT) cell-free infectivity could still transmit via cell-cell contacts and were neutralized by serum from naturally infected cows. These selected HT–BFV variants will shed light into virus transmission and potential routes of intervention in the spread of viral infections. It will also allow the improvement or development of new promising approaches for antiretroviral therapies.

## 1. Introduction

Spumaretroviruses, also known as Foamy Viruses (FVs), were initially discovered in 1954 by Enders and Peebles in primary monkey kidney cell cultures [1]. The name of this retrovirus subfamily is derived from the cytopathic effect (CPE) induced upon their replication in adherent cell cultures of epithelial or fibroblastoid origin [2]. Similar to other retroviruses, FVs contain the canonical *gag, pol,* and *env* structural genes. They also contain additional open reading frames under the control of the 5′-long terminal repeat (LTR) and an internal promoter located in the 3′-end of the *env* gene [3,4]. As an additional distinct feature of FVs, the *pol* gene is encoded by a spliced mRNA. Furthermore, FVs release non-infectious Env-only subviral particles and there is a strict dependence on capsid-glycoprotein interactions for virion release from the cells [5,6,7]. These and other unique features of FVs may be related to their unconventional gene expression and replication strategies, and a long FV-host co-evolution [2,8]. FVs are widespread among non-human primates, bovines, felines, and equines [9,10]. Due to the apparent lack of pathogenicity and their broad tissue tropism, FVs are promising vectors for gene and vaccine antigen delivery [5].

Bovine foamy virus (BFV, also known as bovine syncytial virus) is a member of the understudied non-human Spumaretrovirus subfamily and was first isolated from cattle in 1983 [11]. Though there is no obvious disease associated with BFV infection, there is a high prevalence of BFV in cattle [12,13]. In addition, there is a potential for zoonotic transmission of BFV, since it is detectable in the human food chain through raw milk [13,14,15]. Genomic analyses revealed similar sequence properties between BFV and the other FVs, as well as a compatible phylogenetic position [10,16,17]. Four BFV isolates from the United States (GenBank accession number NC001831.1) [16], China (accession number AY134750.1) [18], Poland (accession number JX307861) [19], and Germany (accession number JX307862) [20] are currently known. Phylogenetic analyses of all four BFV isolates demonstrate a grouping of the isolates from China and USA, while isolates from Poland and Germany form the European clade [20]. The BFV-Riems isolate used here was first described in 1978 in East Germany [21,22].

Unlike orthoretroviruses, FV particle budding requires the co-expression of Gag and Env and depends on specific interactions between the capsid and the N-terminal Env leader protein Elp [6,7]. Known FV Gag proteins lack a classical myristoylation-membrane targeting signal inherent to orthoretrovirus Gag proteins and FV Gag is not released as Gag-only subviral particles (SVP). Biophysical analysis of the capsid structure by cryo-electron microscopy and surface plasmon resonance suggests that a direct and specific binding between the Elp subunit of Env and the N-terminal region of Gag is vital for virion release [7,23]. Retrovirus Gag frequently associates with cellular membranes via Gag myristoylation. In human immunodeficiency virus (HIV)-1, for instance, the N-terminal region of the Gag matrix (MA) subunit contains a myristoylation motif that is covalently modified by myristate, a 14-carbon saturated fatty acid [24]. The attachment of myristate is catalyzed by cellular N-myristoyltransferase (NMT), which uses myristoyl-coenzyme A (CoA) as the active substrate [25]. It was reported for primate/prototype FV (PFV) and feline FV (FFV) that the essential Gag-Env interactions can be replaced by artificial N-terminal fusion of heterologous membrane targeting signals to Gag *in vitro*, leading to Env-independent SVP release [26,27,28]. Alternative Gag membrane re-targeting mechanisms have been also employed for PFV [29].

Viral transmission from infected to non-infected cells occurs by cell-free transmission, involving the release of particles into the extracellular space, or cell-cell contact [30,31]. Viruses have evolved to utilize and manipulate different modes of transmission. For instance, some alphaviruses are highly infectious as cell-free particles and a single virion can enter a cell and cause infection [32,33]. Other viruses, such as human T cell lymphotropic viruses (HTLV-1 and HTLV-2), are predominantly transmitted via cell-cell interactions [34]. Retroviruses, such as HIV, can spread efficiently by both cell-cell and cell-free modes [35,36]. Unlike all other known FVs, which transmit via both cell-cell and cell-free routes, BFV is highly cell-associated, with undetectable cell-free transmission [21]. The way of viral transmission has important implications on the design of biomedical prevention and intervention, as cell-associated viruses are less susceptible to some approaches than cell-free viruses [36]. Due to its tight cell-association, BFV is an excellent model system to study virus adaption to cell-free transmission and identify the principles of viral transmission. The BFV–Riems isolate sequenced and characterized by our lab is especially suited for such studies, since it is the only BFV isolate consistently propagated in bovine cells *in vitro*, eliminating any effects arising from co-adaptation to heterologous host cells [20].

First, BFV-Riems replication in primary calf trachea cells was examined for BFV gene expression, cyto-pathogenicity, particle release, cell-cell transmission, and neutralization by serum factors. Next, we showed that the BFV-Riems budding machinery is similar to that of other FVs. This led us to select for BFV variants with enhanced cell-free transmission in bovine and non-bovine cultures by serial passaging. Interestingly, cell-free high titer (HT) BFV still transmits by cell–cell transmission and is neutralized by serum from naturally infected cows. The experimental system described here will extend our basic knowledge on FV particle release and viral transmission. This knowledge may contribute to the optimization of vectors for applications in cancer gene- or immunotherapy. A better understanding of virus transmission may also enhance our ability to intervene with the spread of viral infections and enable improvement or development of promising novel approaches for antiretroviral therapies.

## 2. Materials and Methods

### 2.1. Virus and Cell Culture

The BFV strain BFV-Riems [21] was obtained from the Friedrich Löffler-Institut (FLI) in Riems, Germany. The virus was propagated in primary calf trachea cells (KTR; Roland Riebe, FLI), and used as wild-type virus throughout this study. HEK293T human epithelial kidney cells were used for transfection assays. These cells were grown in 10 cm dishes in Dulbecco's Modified Eagle Medium (DMEM) supplemented with 10% fetal calf serum (FCS) and 1% penicillin-streptomycin solution.

The baby hamster kidney cell line BHK-21 (ATCC CCL-10) and Madin-Darby bovine kidney (MDBK) cells were grown in DMEM supplemented with 10% fetal horse serum (FHS) and 1% penicillin-streptomycin solution and used for cell-free BFV serial passaging and selection. The BFV indicator cell line (BICL) and MDBK indicator cell line (MICL) for BFV were derived from BHK and MDBK cells, respectively, and contained a green fluorescent protein (Gfp) expression cassette under the control of the BFV long-terminal repeat (LTR) promoter [37]. Both were cultured in DMEM supplemented with 10% FHS, 1% penicillin-streptomycin and 200 µg/mL G418 and used for titration of wild-type (wt) BFV and selected variants.

### 2.2. BFV Propagation, Titration and Neutralization Assays

The original BFV-Riems isolate was initially propagated by co-cultivation of infected and uninfected KTR cells. Non-adherent cells were removed after 24 h and the cultures were passaged at 1:3 ratios until massive CPE appeared. Once CPE was evident, the cells were harvested or, for propagation of the virus, fresh cells were added.

To test the influence of serum on BFV produced in bovine KTR cells, infected cells were propagated in DMEM supplemented with 0%, 1%, and 10% of FCS, FHS, Serum Replacement Solutions 1, 2, and 3 (Sigma, Munich, Germany), and Serum Replacement Solution (PeproTech, Hamburg, Germany), according to the manufacturer’s instructions, or in OptiMEM (Life Technologies, Darmstadt, Germany).

BFV was adapted to use cell-free transmission in MDBK and BHK-21 cells by serial passaging using cell-free culture supernatants rather than cell co-cultivation. Aliquots of 6 × 10^6^ BFV-infected KTR cells and supernatant were frozen in liquid nitrogen and thawed on ice for 30 min. This process was repeated three times. After removal of cell debris by centrifugation at 3000 rpm for 10 min, the resulting virus supernatants were filtered through 0.45 μm filters and used to infect new MDBK or BHK-21 cells. Supernatants from 80%–100% infected cells were filtered and added to new target cells during serial BFV passaging and in vitro evolution.

BFV titers were determined using MICL or BICL cells. Indicator cells were seeded in 96-well plates (1 × 10^4^ cells/well). After 2 h, BFV-containing supernatants were serially diluted 1:6 on the cells. After five days, the supernatants were replaced by phosphate-buffered saline (PBS). BFV titers were determined by life cell fluorescence microscopy for Gfp signals (Leica DM IL LED, Wetzlar, Germany).

For serum neutralization assays, late passage HT–BFV derived from BHK-21 or MDBK cells was serially diluted from 10^4^ to 10^1^ fluorescent focus forming units (FFU)/mL and incubated with serially diluted cow sera 11 and 33 (kindly provided by Magdalena Materniak and Jacek Kuzmak, PIWet, Pulawy, Poland) for 30 min and subsequently inoculated on sub-confluent MICL and BICL cells. The inoculum was replaced after 4 h and the cultures were supplemented with normal 10% FHS-DMEM. Viral titers were determined after five days by fluorescence microscopy.

### 2.3. DNA Transfection into 293T Cells

The day before transfection, 293T cells were seeded at a density of 20%–30%. The next day, cells were transfected with plasmid DNA by using the calcium phosphate (CaPO_4_) method. In a 10 cm dish, a total amount of 10 μg plasmid DNA was diluted in a total volume of 450 µL double-distilled water (ddH_2_O). A mixture of plasmid DNA in water and 45 µL of 2.5 M CaCl_2_ was prepared and added dropwise to 450 µL of 2 x HEPES-buffered saline (50 mM HEPES, 280 mM NaCl, 1.5 mM Na_2_HPO_4_, pH 7.12) under constant gentle shaking [28]. The mixture was incubated at room temperature for 30 min and added dropwise to the cells. After incubation for 12 h at 37 °C and 5% CO_2_, the medium was replaced with fresh growth medium and the cells incubated for an additional 36 h.

### 2.4. Purification of wt and Sub-Viral BFV Particles

To isolate viral particles, 10 mL cell culture supernatants containing BFV particles or SVPs were cleared by centrifugation for 10 min at 405 × g. Subsequently, 7 mL of each supernatant was layered on a 2 mL cushion of 20% sucrose in PBS (w/v) and separated by ultracentrifugation in a SW41Ti rotor (Beckman Coulter, Krefeld, Germany) at 4 °C for 2 h at 28,000 rpm. After removing the medium and sucrose, the non-visible pellet was resuspended in 30 μL 1% SDS in PBS containing complete protease inhibitor cocktail (Roche, Mannheim, Germany) and stored at −20 °C before immunoblotting.

### 2.5. Indirect Immunofluorescence

For indirect immunofluorescence (IIF), BFV-infected KTR cells were seeded on glass coverslips. Five days post-infection (p.i.), the cells were washed with PBS and fixed in ice-cold 100% methanol/0.02% ethylene glycol tetraacetic acid (EGTA). Polyclonal BFV Gag-MA (matrix domain of Gag) specific hyper-immune serum was used as the primary detection serum (diluted 1:1000 in PBS with 3% bovine serum albumin (BSA)). As the secondary antibody, Alexa-594 or Alexa-488-coupled anti-rabbit immunoglobulin IgG antibodies (diluted 1:2000 in PBS with 3% BSA) were used. The respective antibody was incubated for 60 min on the coverslip followed by three 10-min PBS wash steps. For nuclear staining, Hoechst 33342 was used (1:1000 dilution) concurrently with the secondary antibody. Coverslips were finally washed in double-distilled water and ethanol before mounting on glass slides.

### 2.6. Transmission Electron Microscopy

BFV-infected cells were harvested and processed for transmission electron microscopy (TEM) as described previously [38].

### 2.7. Flow Cytometry

BFV-infected MICL cells were harvested at different time points. The cells were washed three times in PBS (405 × g, 5 min) and strained before measurement by flow cytometry. Acquisition was performed using a Becton Dickinson FACSCalibur (BD Biosciences, Heidelberg, Germany) and data analyzed using FlowJo^®^ software (FlowJo LLC, Ashland, Oregon, USA).

### 2.8. DNA Extraction, PCR and Cloning

To extract viral DNA, BFV-infected cells were washed with PBS, transferred into an Eppendorf tube using a cell scraper, pelleted in a micro-centrifuge, and resuspended in an appropriate volume of PBS. DNA was extracted using a DNeasy Blood and Tissue Kit (Qiagen, Hilden, Germany) following the manufacturer's instructions.

To construct eukaryotic BFV expression vectors, BFV *gag* and *env* genes were amplified using the high-fidelity Phusion^®^ DNA polymerase (New England Biolabs, Frankfurt, Germany) using the primers listed in Table 1, 10 ng template DNA from wt and HT-BFV-infected KTR cells using the following conditions. Polymerase chain reaction (PCR) amplification was performed in a Mastercycler (Eppendorf, Hamburg, Germany) in 50 μL using 32 cycles of 95 °C for 15 s (denaturation), 54 °C for 30 s (annealing), and 72 °C for 60 s (elongation). The optimal annealing temperatures were dependent on the melting temperatures of both primers. Before the first cycle, a 2 min denaturing step was performed at 98 °C. A final extension was performed at 72 °C for 10 min to allow for complete amplification.

The cytomegalovirus immediate-early (CMV-IE) promoter-based FFV Gag expression construct pBC-Gag-oPRE was used as backbone for the BFV Gag expression construct, utilizing an optimized post-transcriptional regulatory element (oPRE) from the woodchuck hepatitis virus and a non-coding heterologous sequence providing the splice-donor (SD) and splice-acceptor (SA) sites [39]. FFV *gag* was replaced by PCR-amplified BFV *gag* using *Age*I and *Bam*HI resulting in clone pBC-BFV-Gag-oPRE. The plasmid pBC-Gag-oPRE was subsequently used as the backbone to construct the Gag myr-sub and myr-added clones using *Age*I and *Bam*HI. BFV *env* was cloned to the pBC12 backbone using *Hind*III and *Sma*I.

**Table 1:** PCR primers used for bovine foamy virus (BFV) wt and myr-Gag and Env expression cloning.

Oligonucleotides used for myr-add and -sub mutagenesis 5′-3′	
Gwt-F	CGAGCCACCGGTCCGCCATGGCTCTTAATGACTTCGA
Gwt-R	CGAGCCGGATCCTTAAGATGATTGCCCTTGATTTCCAC
Src-sub	CGAGCCACCGGTCCGCCATGGGC**AGCAGCAAGAGCAAGCCCAAG**GCTCTCCAGGGGTATTTACC
Lck-sub	CGAGCCACCGGTCCGCCATGGGC**TGCGGCTGCAGCAGCCACCCC**GCTCTCCAGGGGTATTTACC
Src-add	CGAGCCACCGGTCCGCCATGGGC**AGCAGCAAGAGCAAGCCCAAG**ATGGCTCTTAATGACTTCGA
Lck-add	CGAGCCACCGGTCCGCCATGGGC**TGCGGCTGCAGCAGCCACCCC**ATGGCTCTTAATGACTTCGA
**Oligonucleotides used to construct Gag and Env expression constructs 5′-3′**	
Gag-F	CGAGCCACCGGT GGAGGAGGTCACAACAATTGG
Gag-R	CGAGCCGGATCCCTCCACGTAATACACATC
Env-F	CGAGCCAAGCTTGGACCTGCCAAAGGTGTGGT
Env-R	GGGCCCTCCTCTAAGTTCATCTCG

Sequences encoding the myr signals are in bold face letters. The *Age*I (ACCGGT), *Hind*III (AAGCTT) and *Bam*HI (GGATCC) restriction enzyme sites are underlined.

### 2.9. Immunoblotting

Cells were washed with PBS, lysed by adding an appropriate amount of lysis buffer (PBS, 1% SDS, 1x Roche complete protease inhibitor) and transferred into an Eppendorf tube using a cell scraper. Genomic DNA was digested with 250 units of Benzonase^®^ Nuclease (37 °C, 20 min). Protein samples were boiled at 95 °C for 1–3 min in SDS loading buffer with containing reducing agent (Fermentas, St. Leon-Rot, Germany). Samples were separated by SDS-PAGE followed by transfer to a nitrocellulose membrane. BFV proteins were detected using polyclonal hyper-immune sera specific against Gag-MA (matrix domain of Gag) or Bet (full protein) (1:5000 dilutions in PBS with 3% BSA). Anti-rabbit IgG conjugated with horseradish peroxidase was used as secondary antibody. Signals were visualized using the enhanced chemiluminescence (ECL) detection system (Amersham Biosciences, Freiburg, Germany). Likewise, BFV-Riems Gag and Bet were detected using bovine reference sera of BFV100-infected cattle (provided by Jacek Kuzmak) and protein G conjugated to horseradish peroxidase as a secondary antibody.

## 3. Results

### 3.1. Virological Characterization of the Original BFV-Riems Isolate 

#### 3.1.1. BFV-Riems Gene Expression in Primary Bovine KTR Cells

**Figure 1:**
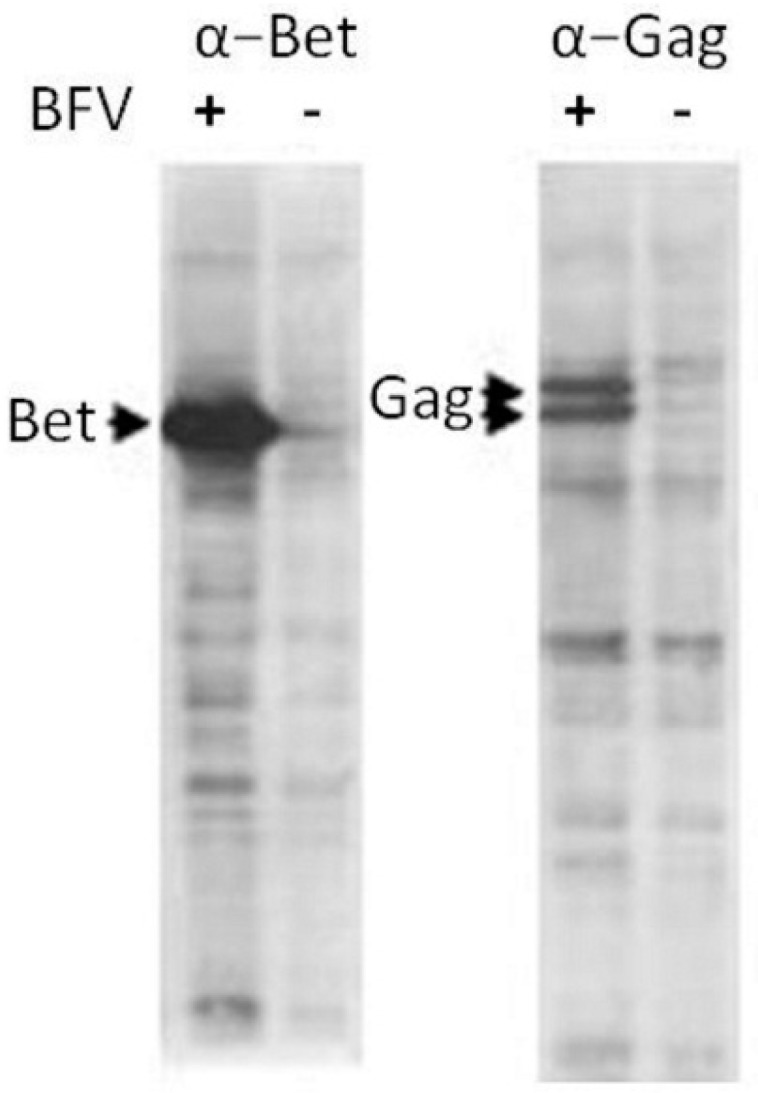
Expression of Bet and Gag in BFV-Riems-infected KTR cells. Cell lysates were harvested from BFV-Riems infected and uninfected KTR cultures for immunoblotting using BFV-Riems-specific antisera directed against BFV Bet and Gag MA. The positions of detected proteins are indicated by arrows.

The original BFV–Riems isolate from KTR cells was characterized for gene expression, particle release, serum neutralization, and transmission. Since the BFV isolates are known to be highly cell-associated and virus propagation is best performed by co-cultivation of uninfected cells with BFV-infected cells [20], BFV-Riems proteins were produced in KTR cells infected by co-cultivation and analyzed by immunoblotting and IIF (see below) using rabbit hyper-immune sera raised again the N-terminal MA domain of BFV Gag. The immunoblot showed robust expression of Gag and canonical C-terminal Gag processing by the BFV protease PR, as typical for BFV (Figure 1). Correct intracellular Gag processing is likely associated with functional Pol expression, capsid assembly, and genomic RNA-mediated incorporation of Pol [40]. The accessory protein Bet is also expressed at comparable levels in BFV-infected KTR cells and confirmed the expected expression profile for the BFV-Riems isolate.

#### 3.1.2. Morphogenesis, Budding and Release of BFV-Riems in KTR Cells

**Figure 2:**
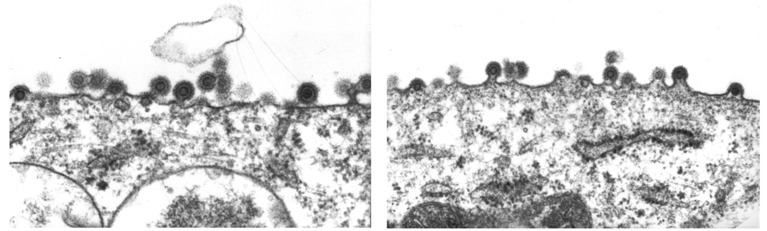
Transmission electron microcopy of BFV-Riems budding at the membrane of infected KTR cells. BFV-infected KTR cells were analyzed in transmission electron microscopy as described previously [38] (performed by Birgit Hub). Two sites of particle assembly from a single BFV-infected cell are shown.

To further explore the tight cell association of BFV, TEM was performed to check for aberrant budding, a tetherin-like phenotype with particle trapping, or other hints of a mechanism hindering efficient infectious particle release. We did not find any evidence for altered particle assembly, release and budding, since both intact and budding particles were observed in almost all images (Figure 2).

#### 3.1.3. Bovine Cell-Based BFV Titration Cells

To sensitively and conveniently monitor BFV infectivity, a new bovine cell-based reporter cell line was constructed. Similar to the BHK-21-based BICL BFV titration cells [37], bovine MDBK reporter cells were constructed. Infection of these cells with BFV is associated with BFV Tas/Bel1 transactivator expression, triggering both viral and Gfp expression, allowing easy and sensitive detection of BFV infection. MDBK cell clones stably carrying a BFV LTR-*gfp* reporter gene cassette were generated using standard methods. A single cell clone, designated MICL, with no basal Gfp expression but strong green fluorescence upon BFV infection, was selected and used for BFV detection and quantification. Titers were determined by measuring fluorescence five days post-infection/titration.

#### 3.1.4. BFV-Riems Infectivity from KTR Cells is Not Inhibited by Bovine or Horse Serum Components

Although fetal sera should not contain substantial amounts of maternal or fetal pathogen-specific antibodies, we analysed whether BFV particles were neutralized by antibodies in the bovine serum used for KTR cell culture. For this purpose, BFV-Riems-infected KTR cells were grown in FCS-containing medium. Cells displaying an obvious BFV-specific CPE were washed and further incubated in medium containing 0%, 1% or 10% of either FCS or FHS, four different serum replacement reagents, or OptiMEM. Infected KTR cells and supernatants were harvested three days after the medium change. Serial dilutions of both cells and supernatants were used to infect sub-confluent BICL and MICL titration cells grown in serum-free OptiMEM medium. After two days, the inoculum was replaced, the cells were washed once and grown for five days in standard cell culture medium before titration by Gfp auto-fluorescence. Data from both titration cell lines show that the different media supplements had variable effects on BFV production and viability of BFV-infected KTR target cells while the supernatants mostly reflected BFV titers in the cell-bound fraction (data not shown). In summary, growth in serum-free cultures did not increase cell-free BFV titers, which strongly implies a lack of BFV neutralization by antibodies in FCS/FHS. Therefore, BFV titers are mainly determined by the overall influence of media on BFV production and cell viability but not by antibodies present in the medium/serum.

### 3.2. Consistent but Low Level Env-Dependent BFV Gag Budding

#### 3.2.1. Low Budding Efficiency of BFV Gag upon Env Co-Expression

**Figure 3:**
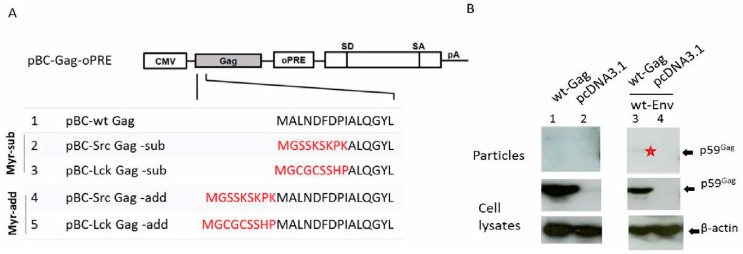
Low levels of BFV Gag-Env subviral particle release. (**A**) Cloning scheme of BFV wt and myr Gag expression clones. Myristoylation signals (marked in red) derived from the human cellular proteins Src and Lck were added onto (-add) or replaced (-sub) part of the N-terminus of BFV Gag in the BFV Gag expression plasmid pBC-BFV-Gag-oPRE. The positions of the CMV-IE promoter, the oPRE, a non-coding heterologous SD and SA site and a poly-adenylation site (pA) are indicated [39]. (**B**) Budding of BFV Gag, alone or together with Env, was analyzed by transfecting HEK293T with the parental wt BFV Gag expression plasmid pBC-BFV-Gag-oPRE alone (lane 1) or together with pBC-BFV-Env (lane 3). The empty vector pcDNA3.1 was used as a negative control (lanes 2 and 4). Two days post-transfection, cell lysates and supernatants were harvested. Equal protein aliquots of cell lysates and subviral particles in supernatant were separated by SDS-PAGE and visualized using a BFV Gag MA antiserum. The position of the Gag precursor (p59) is indicated. The faint band in lane 3 is marked with an asterisk. Proper protein loading was determined by probing for β-actin.

To determine whether the Env-dependence of FV Gag particle release is also valid for BFV, overexpression of Gag and Env of the BFV budding machinery was performed. The BFV structural *gag* and *env* genes were cloned and expressed from sub-genomic expression plasmids and functionally analyzed. BFV *gag* and *env* were amplified via PCR by taking A3 8000 and genomic DNA from BFV-infected KTR as templates, respectively. A3 8000 is a plasmid containing an approximately 8 kb fragment of the BFV genome, from the 5’-end to the middle of BFV genome. This fragment was amplified by long-template PCR taking genomic DNA from BFV-infected KTR as template [20]. FV Gag expression depends on post-transcriptional regulatory elements like an oPRE from the woodchuck hepatitis virus and a non-coding heterologous sequence providing the SD and SA sites [41]. For this reason, the FFV Gag expression plasmid pBC-FFV-Gag-oPRE [39] served as the backbone. The FFV *gag* gene was replaced by the corresponding BFV sequences using standard cloning procedures (Figure 3A). BFV *env* was cloned into the pBC12 backbone using *Hin*dIII and *Sma*I.

The individual contributions of wt Gag and Env of the original BFV-Riems isolate to budding were investigated *in vitro* by co-transfection into HEK293T cells. Two days post-transfection, cell lysates and supernatants were collected for immunoblotting. Figure 3B shows that Gag was expressed in equal amounts with and without Env. However, there was no particle release with Gag alone and very little budding when Gag and Env were co-expressed (faint band in lane 3, marked with an asterisk). The data confirm that BFV directs low-level Env-dependent Gag particle release. However, the efficiency is much lower than those observed in PFV and FFV [5,28,39].

#### 3.2.2. Env-Independent Budding of Myristoylated BFV Gag

**Figure 4:**
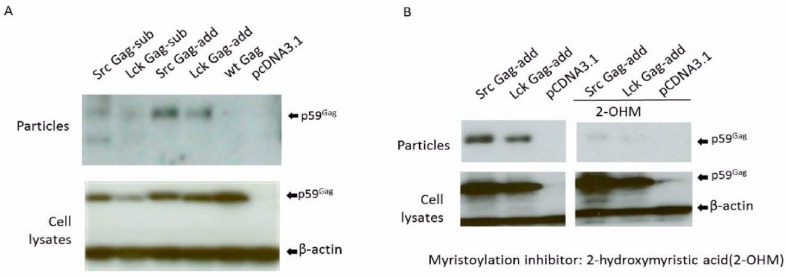
BFV myr-Gag release as subviral particles without Env depends on Gag myristoylation. Proteins of cell lysates and virus particle preparations were harvested two days post-transfection. Equal protein aliquots of cell lysates and SVPs in supernatants were separated by SDS-PAGE and assayed for BFV Gag by immunoblotting. The position of the precursor form of BFV Gag (p59) is marked. Proper protein loading was determined by probing for β-actin. (**A**) The influence of myr-signals on Env-independent BFV Gag SVP release was determined by transfecting HEK293T with the BFV myr-sub or -add Gag expression clones pBC-SrcGag-sub, pBC-LckGag-sub, pBC-SrcGag-add, and pBC-LckGag-add. HEK293T were also transfected with the unmodified parental BFV Gag expression constructs pBC-wtGag. The empty vector pCDNA3.1 was used as a negative control. (**B**) BFV myr-Gag subviral particle (SVP) release is abrogated by the N-myristoyltransferase (NMT) inhibitor 2-hydroxymyristic acid. HEK293T cells were transfected with myristoylated Gag plasmids pBC-SrcGag-add and pBC-LckGag-add with and without NMT inhibitors. The empty vector pcDNA3.1 was used as a negative control.

The Env-dependency of FV Gag budding can be overcome in PFV and FFV by an artificially introduced N-terminal Gag myristoylation signal [26,27,28]. Thus, myristoylation signals derived from human Src and Lck proteins were appended to or inserted into the N-terminus of BFV Gag. For this purpose, the BFV Gag expression plasmid pBC-BFV-Gag-oPRE was used as the backbone to construct the Gag myr-sub and -added clones. BFV myr-Gag sequences were amplified by PCR using modified primers, digested with *Age*I and *Bam*HI and ligated into the correspondingly digested plasmid pBC-BFV-Gag-oPRE (Figure 3A). The efficiency of Env-independent myr-Gag SVPs release was investigated by transfection of HEK293T cells with myr-sub and -add Gag plasmids and analysis of particle release. Due to the absence of BFV Pol in BFV Gag SVPs, Gag is not processed, as shown by the presence of the precursor p59 Gag. As seen in Figure 4A, myr Gag constructs had moderately reduced levels of Gag protein expression compared to wt Gag. Interestingly, even low concentrations of myr Gag restored Gag SVP release in the absence of Env and enhanced budding for myr-add Gag was observed while inserting the myr signal into the N terminus of Gag yielded only marginal particle release. These data are in line with studies in PFV and FFV [26,27,28]. In summary, BFV myr-Gag can restore Gag SVP release without cognate Env. Enhanced budding of myr-add Gag over myr-sub Gag was also demonstrated.

The myristoylation-dependency of the myr-Gag SVP release (Figure 4A) was confirmed by incubating pBC-SrcGag-add- and pBC-LckGag-add-transfected HEK293T from 10 h post-transfection for about 30 hrs with the NMT inhibitor 2-hydroxymyristic acid (2-OHM). No obvious toxicity from 2-OHM was detected during this time. The overall expression of myr-add Gag clones was not affected by the presence of 2-OHM (Figure 4B). As reported in previous studies with FFV [28], release of BFV myr Gag SVPs was dramatically reduced in the presence of 2-OHM, while overall Gag protein expression was not affected. This result indicates that SVP release is not simply due to the attachment of the myr sequence but relies on the myristoylation of Gag. Thus, the myr sequence serves as a membrane-targeting signal and is modified by myristate during particle release.

In summary, the data indicate that the BFV Gag-Env assembly and budding machinery is comparable to that of the other FVs. However, release of Gag-Env and myr-Gag BFV SVPs is intrinsically much less efficient than in PFV and FFV [26,27,28].

### 3.3. Selection of High Titer Cell-Free Infectious BFV (HT–BFV) by Serial Passaging

#### 3.3.1. Selection of BFV with Enhanced Budding Capability and HT Cell-Free Infectivity

Since the particle budding machinery of the original BFV-Riems isolate showed similarity to that of other FVs, we aimed to select HT cell-free infectious BFV variants (HT–BFV) *in vitro*. Using the advantage of the rapid evolution of RNA viruses, including retroviruses, a stringent *in vitro* evolution and selection schedule by serial cell-free passaging of BFV was applied. Selection was performed in parallel in bovine (MDBK) and non-bovine, BFV-permissive hamster cells (BHK-21) to compare differences in the adaptive process and potentially distinguish between co-adaptations to the different host cell species and the HT cell-free transmission phenotype. Selection in the two cell lines was performed in duplicate by two persons to examine the reliability and applicability of the selection procedure.

As starting material, KTR-associated BFV particles were harvested by repeated cycles of freezing and thawing of cells and culture supernatants. Lysates were cleared by low-speed centrifugation, filtered through a 0.45 µm filter, and applied to MDBK and BHK-21 cells. Under these conditions, infected cells were initially split several times before BFV-specific CPE appeared. From cell cultures with enhanced CPE in more than half the cells or more than 80% Gag positivity, as determined by IIF, supernatants were harvested, cleared by low speed centrifugation, and filtered through a 0.45 µm filter before inoculation of new MDBK and BHK21 target cells. During serial passaging, cell-free infectivity was determined using the BFV indicator cell lines MICL and BICL cells, respectively.

**Figure 5:**
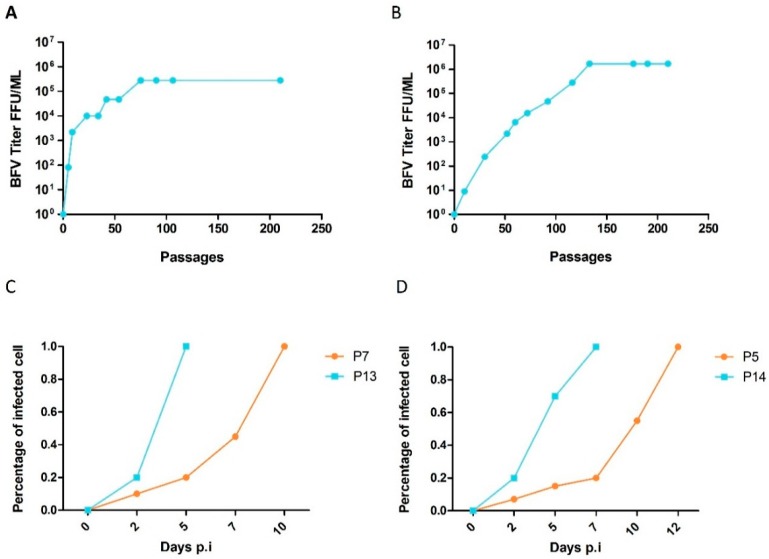
**Kinetics of adaptation of cell-free infectious BFV in MDBK and BHK-21 cultures.** Cell-free infectivity of selected BFV was monitored during serial passaging. **(A-B)** Culture supernatants from serially passaged BFV-infected (**A**) MDBK and (**B**) BHK-21 cells were filtered and titrated on MICL and BICL cells, respectively. Titers are expressed as focus-forming units per ml (FFU/ml). **(C,D)** Percentages of BFV Gag-positive cells as determined by IIF for early passages (P) of BFV from (**C**) MDBK and (**D**) BHK-21 cells were determined at different time points. BFV-infected MDBK cells: P7 and P13; BFV-infected BHK-21 cells: P5 and P14.

BFV with enhanced, HT cell-free transmission was obtained in both culture replicates of both cell lines. The capacity for cell-free transmission dramatically accelerated, especially during the initial passages. Cell-free infectivity increased from almost 0 FFU/ml to levels plateauing at about 10^6^ FFU/mL for BHK-21 cells and 10^5^ FFU/mL for MDBK cells (Figure 5A,B). The time required for complete infection of target cultures decreased with increasing passage numbers (Figure 5C,D). The lower titers of the MDBK-adapted BFV may be due to more efficient innate and intrinsic immunity of these cells. This can be, for instance, through additional barriers to infection, such as intact interferon signaling and subsequent induction of restriction factors [2]. Initial cell-free passages required nearly one month, while the time required for each individual passage decreased to only a few days in the late passages, indicating an increased rate of viral replication. Comparisons between the cell lines were particularly interesting, as HT–BFV required more time and a higher number of passages to reach a plateau in cell-free transmission in BHK-21 cells than in MDBK cells. This correlated with the hypothesis that the viruses had to adapt not only to efficient cell-free transmission but also to the heterologous hamster host cells.

**Figure 6:**
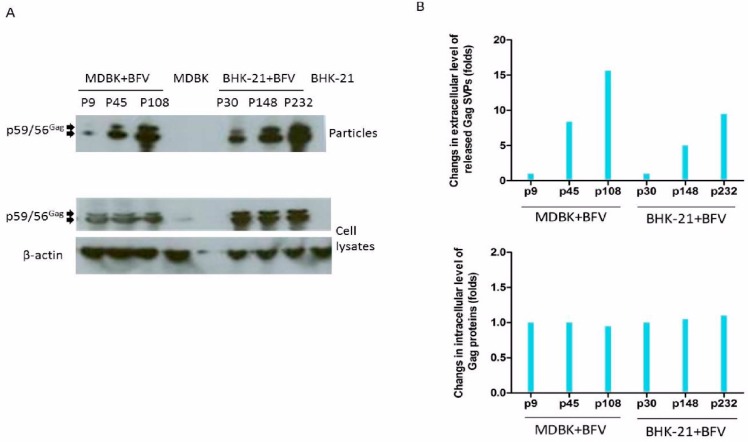
Enhanced budding of late-passage BFV as determined by immunoblotting. (**A**) Cells and supernatants (bottom and top panels) were harvested at the indicated passages for immunoblotting (BFV-infected MDBK cells: p9, p45 and p108; BFV-infected BHK-21 cells: p30, p148 and p232). The Gag precursor (p59) and cleaved form (p56) are marked by arrows. (**B**) Densitometric quantification of relative Gag expression levels (bottom) and budding capacities (top) of BFV from different passages and cell lines as indicated. ImageJ was used for densitometric analysis to quantify the density of the immunoblot bands. The results from the loading controls (β-actin) were used to scale the values of the protein of interest (Gag). Densitometric data of the middle and late passages were normalized to the corresponding values of early passages (p9 and p30, respectively) showing similar intracellular Gag expression levels (bottom panel) but an increased budding capacity with continued selection (top panel).

To characterize the selected HT–BFV variants for particle release, sucrose cushion-enriched supernatants and cell lysates from selected early, middle, and late passages of BFV-infected MDBK and BHK-21 cells were analyzed by immunoblotting. The results clearly showed a gradual increase in particle release with increasing passage number but no major variation in intracellular steady-state levels of the BFV Gag protein in both cell systems (Figure 6A). Densitometric quantification of the relative Gag expression levels and budding capacities of selected BFVs showed similar intracellular Gag expression levels (bottom panel), while the budding capacity increased as the selection continued (top panel) (Figure 6B).

**Figure 7:**
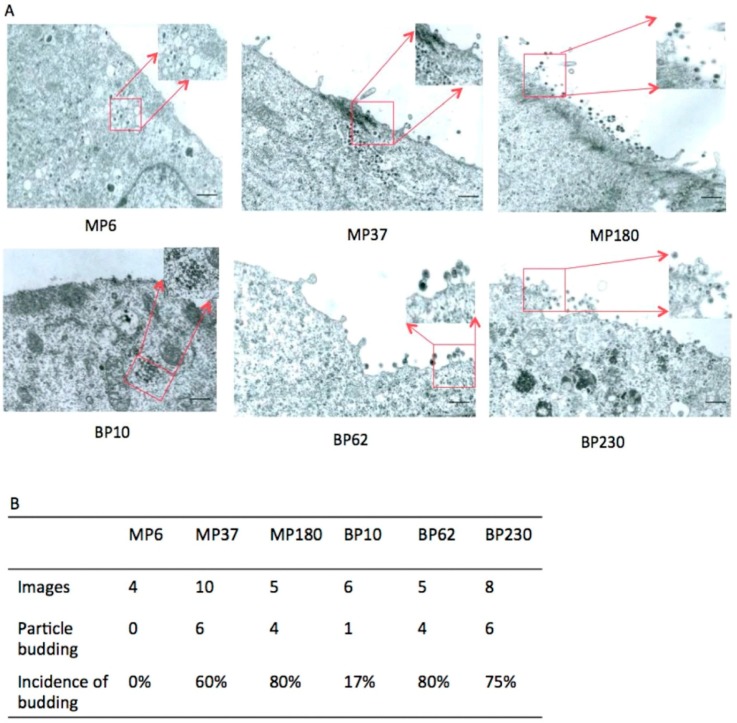
Enhanced budding of late-passage BFV determined by TEM. TEM was performed to directly examine particle release of BFV from different passages. (**A**) BFV-infected MDBK and BHK-21 cells from the indicated passages (BFV-infected MDBK cells: MP6, MP37, and MP180; BFV-infected BHK-21 cells: BP10, BP62, and BP230) were washed three times, fixed for 10 min at room temperature, and processed as described previously [38]. Some views with BFV capsids indicated by red boxes are magnified. Black bars represent 4 µm. (**B**) Quantification of BFV budding from different passages. Different TEM images were used to quantify budding. Only cells with intracellular, clearly visible BFV capsids were scored for the presence of budding events at the cell surface.

To further examine HT–BFV particle release, BFV from several passages, MP6, MP37, and MP180 from MDBK cells (passages of BFV-infected MDBK cells: P6, P37, and P180, respectively), and BP10, BP62, and BP230 from BHK-21 cells (passages of BFV-infected BHK-21 cells: P10, P62, and P230, respectively) were analyzed by TEM. Capsids embedded in electron-dense material were frequently observed in the cytoplasm in early and middle passage HT–BFV. However, extracellular virions were rarely detected in the early samples, while moderate numbers of particles were observed in the middle passages. In the late passages, budding and bursts of viral particles were readily observed seen at the plasma membrane of HT–BFV-infected cells. The results demonstrated an increase in particle release with BFV selection (Figure 7A). To validate this observation, budding was quantified by analyzing several independent TEM images and calculating the incidence of budding. For this purpose, only cells with clearly visible intracellular assembled BFV capsids were scored for the presence of budding events at their plasma membrane. Particle release was rare in early passages but more frequently detectable (more than 50% capsid-positive cells also display plasma membrane budding) in middle and late passages (Figure 7B). TEM analysis thus clearly supports the immunoblotting data showing that HT–BFV displays enhanced budding efficiency. In addition, no substantial differences were observed between HT–BFV selected from MDBK and BHK-21 cells.

**Figure 8:**
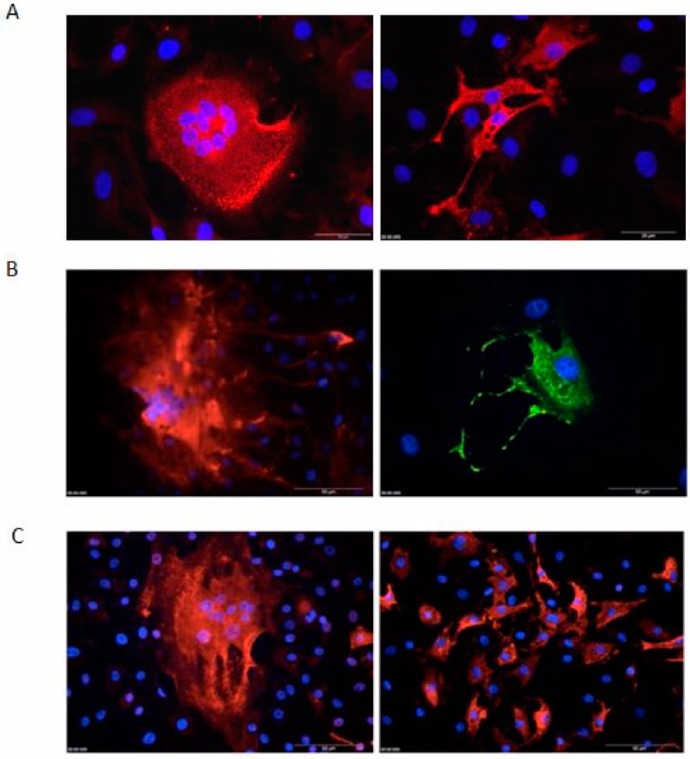
Syncytia, “filopodia-like” structure in BFV, and loss of syncytia formation by serially passaged HT–BFV as determined by indirect immunofluorescence (IIF) microscopy. BFV-infected KTR cells were fixed with ice-cold methanol/EGTA and incubated with a rabbit antiserum against BFV Gag MA. Alexa-594 (red) or Alexa-488 (green)-coupled anti-rabbit IgG was used as secondary antibody. Nuclei were counter-stained with Hoechst 33342 (blue). (**A**) Syncytia of original BFV Riems-infected KTR cells by IIF against Gag. The bars are 20 µm in length. (**B**) “Filopodia-like” structures in original BFV Riems-infected KTR cells detected via IIF. The bar is 50 µm in length. (**C**) BFV-infected KTR cells using the original BFV–Riems isolate (left hand panel) and serially passaged BFV (HT–BFV from passage 30 in BHK-21 cells, right hand panel) were analyzed by IIF against Gag MA. BFV Gag MA (red) and nuclear Hoechst 33342 (blue) staining allowed clear detection of loss of syncytia formation upon serial passaging. The bar is 50 µm in length.

Formation of multinuclear syncytia is a common feature of FVs in many infected cultures. In BFV-permissive KTR cell, FV-specific syncytia are easily detectable by phase contrast microscopy (data not shown, see e.g. [11,21]) and IIF with nuclear co-staining. Thus, original BFV Riems-infected KTR cells were assayed by IIF using Gag MA as a BFV-specific marker. Syncytia characterized as enlarged cells containing several nuclei were readily observed in infected KTR cultures (Figure 8A). In addition, a potential virus spread by “filopodia-like” cell-cell contacts was frequently detected, often resulting in localized clusters of BFV-positive cells (Figure 8B).

Since we detected a loss of syncytia formation of selected HT–BFV at passage 18 in MDBK and passage 24 in BHK-21 cells, we examined whether loss of syncytia induction was also detectable in KTR cells. Antigen expression, subcellular distribution, and syncytia formation in KTR cells infected with the original BFV-Riems isolated by co-cultivation and KTR cells infected by cell-free BFV particles from passage 30 in BHK-21 cells were analyzed by IIF using Gag MA antiserum. While Gag expression and sub-cellular localization did not change dramatically, BFV-specific CPE of large multinucleated giant cells was completely lost at higher passage numbers (Figure 8C).

#### 3.3.2. HT–BFV Can Still Be Neutralized by Sera from BFV-Infected Cows

To examine whether the serological reactivity of HT-BFV was grossly altered by the selection process, serum neutralization assays of HT-BFV using sera from naturally BFV-infected cows were conducted. Sera 11 and 33 from naturally BFV-infected cows specifically detected BFV Gag and Bet proteins by immunoblotting (data not shown) and were selected for the neutralization assays. Late-passage HT–BFV derived from BHK-21 and MDBK were serially diluted from 10^4^ to 10^1^ FFU/mL, incubated with serially diluted cow sera 11 and 33 for 30 min, and inoculated on sub-confluent MICL and BICL cells. The inoculum was replaced after 4 h and the cultures supplemented with normal 10% FHS-DMEM. Titers were determined after five days by fluorescence microscopy. Both HT-BFV were neutralized by sera from BFV infected cows 11 and 33 (Table 2) using HT–BFV from MDBK and BHK-21 cells. The results confirm that the HT cell-free transmission of the selected viruses was not due to a gross escape from serum neutralization or dramatic antigenic changes.

**Table 2:** Neutralization of high titer (HT)-BFV by serum from BFV-infected cows 11 and 33.

**HT-BFV derived from BHK-21**	**Normal medium**	**Serum from BFV naturally infected cows 11**					**Serum from BFV naturally infected cows 33**				
FFU/ml	100 μl	100 μl	33 μl	11 μl	3.6 μl	1.2 μl	100 μl	33 μl	11 μl	3.6 μl	1.2 μl
10^4^	++++	+++	++++	++++	++++	++++	+++	++++	++++	++++	++++
10^3^	+++	+	+	++	++	+++	+	++	++	++	+++
10^2^	++	-	-	-	+	++	-	-	-	++	++
10^1^	+	-	-	-	-	+	-	-	-	-	+
**HT-BFV derived from MDBK**	**Normal medium**	**Serum from BFV naturally infected cows 11**					**Serum from BFV naturally infected cows 33**				
FFU/ml	100 μl	100 μl	33 μl	11 μl	3.6 μl	1.2 μl	100 μl	33 μl	11 μl	3.6 μl	1.2 μl
10^4^	++++	+++	+++	++++	++++	++++	+++	++++	++++	++++	++++
10^3^	+++	-	+	++	++	+++	+	++	++	++	+++
10^2^	++	-	-	-	+	++	-	-	-	++	++
10^1^	+	-	-	-	-	+	-	-	-	-	+

(++++), (+++), (++), (+), relative number of GFP-positive cells; (-), no GFP autofluorescence.

#### 3.3.3. HT-BFV Can Spread via Cell–Cell and Cell-Free Transmission

To determine whether HT–BFV can still spread via cell–cell contact, the efficiencies of cell-free and cell-cell transmission were measured. It was important to establish experimental conditions that allowed an approximate comparison of both modes of transmission. Cell-free transmission is more effort- and time-intensive than cell-cell transmission. Early after a new infection, viral infection of target cells is mediated mainly by cell-to-cell spreading, with little or no contribution from cell-free viral transfer, which is significantly less efficient [33,36,42]. Based on this information, we compared new infections in both infected cell co-cultures and cultures infected exclusively with cell-free supernatants.

**Figure 9:**
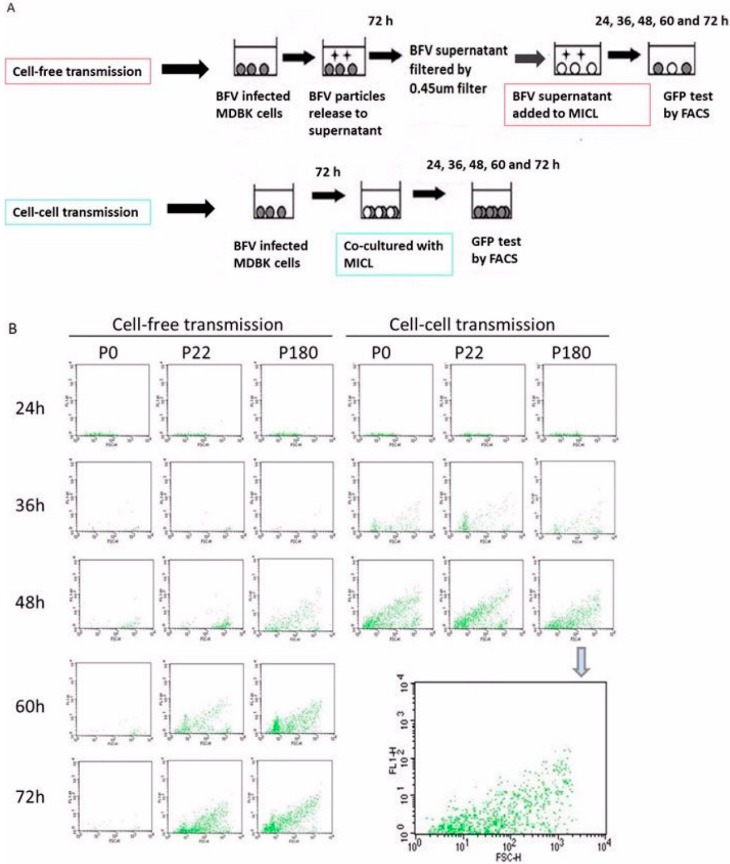
Cell–cell transmission is retained in HT-BFV. The ability of HT-BFV to spread by cell–cell transmission was examined using the original BFV-Riems and HT-BFV from different passages in MDBK cells. (**A**) Experimental approach comparing BFV transmission by cell-free virus or by spread via co-cultivation (modified according from [33]). Original BFV–Riems and BFV from middle (p. 22) and late passages (p180) were cultured in MDBK cells in 10 cm dishes. When more than 80% of the cells were infected, cells were cultivated with MICL cells at a ratio of 1:6. The corresponding supernatants were filtered using a 0.45 µm filter and applied to target cells for cell-free transmission. Infection was measured at different time points (24 h, 36 h, 48 h, 60 h, and 72 h) by detecting BFV Tas-activated Gfp expression using flow cytometry. Cell-free transmission is indicated by a red box. Cell-cell transmission is indicated by a blue box. (**B**) Representative result of BFV infection via cell–cell and cell-free transmission. BFV-infected MICL cells were harvested at different time points. Cells were washed thrice in PBS and filtered before flow cytometry using a BD FACSCalibur flow cytometer. The enlarged panel at the right side represents data from cell-free transmission of passage P180 at 48 h post-infection.

Figure 9A schematically shows the experimental approach used in this study (modified according to [33]). Filtration through a 0.45 µm filter, which allows continuous passaging of viral particles but not cells, was used here to establish cell-free infections. Virus particles and BFV-infected cells from early, middle, and late passages were derived from MDBK cells and applied to MDBK-derived MICL BFV reporter cells either by cell–cell or cell-free infections. The simultaneous use of both MDBK and MICL cells facilitated direct and efficient monitoring of new infection events. The amount of filtered supernatants correlated with the number of infected cells. Importantly, the ratio of infected to target cells was 1:6, allowing the measurement of infectivity within a reasonable time and allowing discrimination between the two transmission routes. This approach cannot exclude the contribution of cell-free transmission in cell co-cultures late after infection, since co-cultures allow a combination of cell-free and cell-cell spreading after long-term infections. Therefore, it was extremely important to monitor early time points after infection. Here, BFV infection was measured directly after a new infection at different time points: 24, 36, 48, 60, and 72 h post-infection by flow cytometry to detect Gfp expression in newly-infected MICL cells.

At 24 h post-infection, BFV infection by cell-free and cell-cell transmission was barely visible (Figure 9B). At 36 h post-infection, a clear Gfp signal was observed in co-cultures from different passages but cell-free infection was still not above background. This demonstrates that efficient cell–cell transmission, but not cell-free spread, is detectable in the initial phases of a new infection by BFV from different passages. These data clearly show that HT–BFV from late passages still maintains the ability to spread by cell-cell transmission. By 48 h post-infection, an increase in infectivity was observed by both transmission routes for *in vitro* selected BFV while the original BFV–Riems only spread via cell–cell transmission whereas cell-free transmission was almost undetectable throughout the whole time frame. As expected, BFV from late passages had better cell-free infectivity at 48 h compared to those from passage 22. Moreover, more time was required for complete infection of the culture by cell-free spread (70 h) than by cell-cell transfer (48 h). Overall, the data confirm a lack of cell-free transmission for the original BFV–Riems, while BFV with increased cell-free infectivity retained its ability to spread via cell–cell transmission.

## 4. Discussion

In the current study, we set out to characterize and functionally analyse the BFV–Riems isolate in infected cultures with special emphasis on the low or almost non-existing cell-free virus transmission of all known BFV isolates *in vitro*. Unlike the simian and feline FVs, BFV spreads pre-dominantly by cell-cell transmission and “filopodia-like” structures (shown here in primary, non-transformed and not immortalized bovine KTR cells) may be utilized for BFV transmission. By using a rigorous *in vitro* evolution procedure, HT cell-free transmitted BFV was selected and characterized with special emphasis on gene expression, gross antigenic changes, and mode of transmission.

The inefficient cell-free transmission of all known BFV isolates in cell cultures may be due to the non-permissiveness or reduced permissiveness of the target cells used *in vitro*, the inefficient particle release, the consequence of antibody-mediated immune responses, or anti-viral restriction factors [43,44,45]. By using different methods, an extremely low or even a lack of releasing infectious particles of the BFV–Riems isolate was confirmed while intact particles and their apparent budding at the cell membrane were readily observed. In addition, an Env-dependent budding of BFV Gag-Env SVPs was detectable at very low levels upon overexpression of both proteins in human HEK293T cells and the Env-independent release of engineered BFV myr-Gag proteins was shown to be blocked by the myristoylation inhibitor 2-OHM. Both features are comparable with the situation in PFV and FFV [26,27,28], confirming that the overall budding and release machinery of BFV exhibits the known FV-specific peculiarities. The possibility that BFV neutralization by antibodies in the fetal bovine and equine sera used for cell culture is interfering with efficient cell-free virus spread was explored and excluded since such effects were not detectable under the conditions used here. This finding is in line with the fact that the bovine and equine placenta are of the epitheliochorial type preventing the transfer of maternal antibodies to the fetus and thus the fetal serum [46]. However, since target organs or target cells for either productive replication and/or persistent infection are largely unknown for any of the known non-primate FVs [10,14,15,20], the possibility of using inappropriate target cells in *in vitro* cell cultures cannot be formally excluded at present. In the current study, bovine non-transformed, mortal KTR (calf trachea), hamster BHK-21 (Baby hamster kidney fibroblast) and MDBK (Madin-Darby bovine kidney) cells were analyzed. The wt BFV-Riems isolate showed almost exclusively cell-to-cell transmission in all these cells, making a major effect of low level permissiveness of the target cells used here unlikely. In addition, FVs are considered to have a very broad range of permissive target cells, at least *in vitro* [10]. Finally, the detrimental effects of anti-viral restriction factors cannot be excluded at present. Importantly, the *in vitro* evolution screen used here did not select host cells with a higher permissiveness towards BFV but only the serially passaged BFV-Riems isolates grown in the two unselected, parental cell lines. The selection was performed in bovine and hamster cells in parallel. It is especially noteworthy that comparisons between the cell lines showed that the titer of MDBK-selected BFV accelerated more rapidly in the initial period compared to the BHK-21-derived BFV. Similarly, HT cell-free BFV selection required more time and passages to reach a plateau in BHK-21 cells than in MDBK cells. This finding supports the view that BFV required additional genetic alterations to adapt to the heterologous hamster target cells. However, the absolute titers of HT-BFV from BHK-21 cells were higher than those from MDBK cells. 

Further virological work will be needed to address questions related to cellular restriction factors. Molecular virus genetics and evolutionary studies are required to address all issues related to alterations in viral functions involved in efficient cell-free transmission [2]. Here, viral proteins counteracting restriction factors or the Gag-encoded late (L) domain are likely candidate targets of adaptive changes enforced by the selection criteria applied to the BFV–Riems isolate.

The viral proteins detected by immunoblotting in the cellular lysates show similar steady-state expression levels throughout the selection procedure, indicating that a simple increase in viral protein synthesis did not play a role when selecting for HT-BFV variants. In addition, we did not detect any gross differences in Gag protein sub-cellular localization, although TEM studies indicate increased budding at the plasma membrane in MDBK and BHK-21 cells. Here, the question emerges whether cytosolic trafficking of preassembled or fully assembled capsids described for PFV is affected by any of the induced adaptive changes [47]. While Gag did not show obvious differences in the assays performed here, we detected a loss of Env-mediated syncytia induction, the prominent cytopathic effect of FV-infected cells, in the cultures after approximately 18 and 24 passages in MDBK and BHK-21 cells, respectively. It is currently unknown whether essential residues of the fusion machinery of the trans-membrane (TM) domain of Env were affected by amino acid changes or whether other, more indirect, mechanisms are involved. A possible scenario would be that changes in the ratio of particle-associated *versus* free Env occurred and that therefore fusion-competent Env is simply no longer present on the surface of HT-BFV-infected cells.

BFV is highly cell-associated in cell culture with no or only miniscule transmission via cell-free virus [11]. The mode of BFV transmission in the natural host and upon possible interspecies transmission is not known. However, it is known that BFV can be isolated from peripheral blood lymphocytes and milk cells of BFV-positive cattle exclusively in a cell-associated fashion [13]. Infectious BFV can be isolated from raw milk cells, implicating the potential risk of interspecies transmission of BFV to humans [14]. Since cell-free isolation of BFV from milk was not successful (our unpublished observations), the situation in BFV is reminiscent of T cell leukemia viruses (HTLV-1 and HTLV-2) which are also known to be predominantly transmitted by infected cells and not cell-free virus particles [33]. Since transmission of cell-associated virus is mediated by cell–cell contacts with recipient cells, a major advantage of cell-cell transmission is the resistance to neutralization by antibodies. In addition, due to high local particle concentrations, transmission is considered to be much more efficient, thereby significantly contributing to the pathogenesis of viral infections [30,48].

The data presented here confirm that BFV is an excellent model system to study *in vitro* molecular evolution of a specific viral function. In this case, we studied HT cell-free virus release and infectivity, though close relatives and likely also the ancestors of BFV carried this trait. The fact that the HT cell-free transmission phenotype was reproducibly acquired twice in two different cell lines indicates that the required molecular building blocks are still present in the original BFV-Riems isolate for further adaptation [49]. It is, however, highly unlikely that the evolution was simply reverted during the *in vitro* evolution screen performed here. Instead, alternative or variant solutions to overcome the selection hurdle (cell-free transmission) have likely evolved. Corresponding molecular evolution studies are currently under way to address all these questions.

While there are many unknowns and speculations concerning the advantages of cell–cell *versus* cell-free virus transmission in pathogenic human and animal viruses, the BFV system, with the option to study virus spread and transmission in cattle and sheep, offers an excellent, manageable, reliable and robust animal model to investigate these issues [50] while, for instance, spread and transmission studies for HTLV-1 and 2 and HIV in primates are not possible for different reasons. It would be very important to know whether infection of cattle or sheep with HT cell-free BFV results in pathology. Information on the risk of disease development due to HT-BFV adaptation to the original and new host is important and will expand our knowledge of FV infections. Alternatively, cell culture HT-BFV may be no longer able to replicate or persist. Unraveling the reasons behind this would shed light on why BFV developed into a tightly cell-associated virus. Immune surveillance may be an additional critical point affecting virus replication and persistence [51]. Comparing the cell culture-selected HT-BFV variant with the original BFV-Riems isolate displaying cell-cell *versus* HT cell-free transmission for virus persistence, spread, molecular evolution, and immune detection or clearance within experimentally infected animals would be an excellent model.

## 5. Conclusions

In the current study, we established BFV variants that gained the ability of HT cell-free transmission using *in vitro* evolution. The HT-BFV variants were characterized with respect to different parameters, such as gene expression, replication features and immunogenicity. Comparative genetic studies at different stages of the adaptive process conducted here should allow identification of the molecular mechanisms required for efficient cell-free retrovirus particle release, a process essential for translational studies in the area of retrovirus vector development. It is also of prime importance in understanding virus transmission and pathogenicity. 
